# Patient-derived xenografts from circulating cancer stem cells as a preclinical model for personalized pancreatic cancer research

**DOI:** 10.1038/s41598-025-87054-z

**Published:** 2025-01-23

**Authors:** Benedikt J. Wagner, Andreas Ettner-Sitter, Nicolas A. Ihlo, Merle Behr, Sebastian Koelbl, Stefan M. Brunner, Florian Weber, Bettina M. Rau, Hans J. Schlitt, Christoph Brochhausen, Rebecca Schoenmehl, Annalena Artinger, Dorothea Schott, Monika Pizon, Katharina Pachmann, Thiha Aung, Silke Haerteis, Christina Hackl

**Affiliations:** 1 Department of Surgery, University Hospital Regensburg, Franz-Josef-Strauss-Allee 11, 93053 Regensburg, Germany; 2 Institute for Molecular and Cellular Anatomy, University of Regensburg, Universitaetsstrasse 31, 93053 Regensburg, Germany; 3 Faculty of Informatics and Data Science, University of Regensburg, Bajuwarenstrasse 4, 93053 Regensburg, Germany; 4 Technology Campus Hutthurm, Deggendorf Institute of Technology, Hochleiten 1, 94116 Hutthurm, Germany; 5 Institute of Pathology, University of Regensburg, Franz-Josef-Strauss-Allee 11, 93053 Regensburg, Germany; 6 Department of General, Visceral and Thoracic Surgery, Academic Teaching Hospital Neumarkt, Nuernberger Strasse 12, 92318 Neumarkt in der Oberpfalz, Germany; 7 Institute of Pathology, Medical Faculty Mannheim, Heidelberg University, Theodor-Kutzer-Ufer 1-3, 68167 Mannheim, Germany; 8 Simfo GmbH, Kurpromenade 2, 95448 Bayreuth, Germany; 9 Labor Dr. Pachmann Bayreuth, Kurpromenade 2, 95448 Bayreuth, Germany; 10 Faculty of Applied Healthcare Science, Deggendorf Institute of Technology, Dieter-Goerlitz-Platz 1, 94469 Deggendorf, Germany; 11 Bavarian Cancer Research Center (BZKF), University Hospital Regensburg, Franz-Josef-Strauss-Allee 11, 93053 Regensburg, Germany

**Keywords:** Pancreatic cancer, Cancer stem cell, Chorioallantoic membrane, Patient-derived xenograft, Personalized medicine, Liquid biopsy, Cancer stem cells, Cancer therapy, Tumour biomarkers, Pancreatic cancer, Stem-cell research

## Abstract

Patient-derived xenografts (PDXs) provide biologically relevant models and potential platforms for the development of treatment strategies for precision medicine in pancreatic cancer. Furthermore, circulating epithelial tumor cells (CETCs/CTCs) are released into the bloodstream by solid tumors and a rare subpopulation—circulating cancer stem cells (cCSCs) – is considered to be responsible for recurrence and plays a key role in metastasis. For the identification of cCSCs, an innovative in vitro assay to generate tumorspheres was established in this study. The number of tumorspheres and CETCs/CTCs was analyzed perioperatively in 25 pancreatic cancer patients. Additionally, an individual in vivo chorioallantoic membrane (CAM) culture system was used to generate PDXs from these tumorspheres. While overall correlations of CETCs/CTCs with clinicopathological parameters did not reach statistical significance, a significant difference in the number of tumorspheres was observed between patient subgroups with lower and higher UICC stages. This finding underscores their potential as biomarkers, providing valuable insights into clinical decision-making and tumor progression. The application of tumorspheres on the CAM successfully established PDXs within 7 days. These xenografts closely resembled the histological features of the primary tumor. Hence, this model represents a novel and fast option for individualized testing of new therapies for PDAC.

## Introduction

According to “Global Cancer Statistics 2020”, a total of 495,773 new cases and 466,003 related deaths were reported globally for pancreatic cancer in 2020, with almost as many deaths as initial diagnoses^1^. Pancreatic cancer is fatal after a short course of therapy in approximately 90% of patients and tumors almost always develop resistance to conventional treatments. Pancreatic cancer is the fourth leading cause of death among all cancers in Germany and the United States and is expected to be the second leading cause of death by 2030^2^. The high mortality rate is mainly caused by metastases leading to dysfunction in vital organs^3^. This makes the improvement of diagnosis and treatment of pancreatic cancer an important medical challenge.

Currently, surgical resection is the only potentially curative option. The goal is to remove the tumor tissue with a safety margin and all involved lymph nodes. The formation of metastases is observed months and years even after complete surgery, indicating that there are certain tumor cells capable of remaining viable in the bloodstream and/or metastases-hosting organs even after several years^4–7^. Current research suggests that disseminated tumor cells in the blood can be a source of cancer metastasis^5,8^. A central question hereby is whether the poor prognosis in pancreatic cancer is due to previously unknown properties of these circulating tumor cells.

Circulating tumor cells derived from solid epithelial tumors can also be referred to as circulating epithelial tumor cells (CETCs/CTCs) which emphasizes their epithelial origin. This origin can be confirmed in the peripheral blood using the epithelial cell adhesion molecule (EpCAM), which is known to be overexpressed in epithelial tumors and assumed to enhance their proliferative and invasive capacities^4^. CETCs/CTCs can be detected in the peripheral blood of cancer patients even at early stages of the disease, offering a non-invasive method for monitoring cancer progression and treatment efficacy^5–8^. In pancreatic ductal adenocarcinoma (PDAC), Plectin-1, a cytoskeletal protein, has emerged as a novel biomarker. Studies have shown that it is overexpressed in PDAC, making it highly specific for identifying CETCs/CTCs in these patients^9^.

However, not all CETCs/CTCs exhibit the same biological behavior. A small subpopulation of these cells, known as circulating cancer stem cells (cCSCs), possesses stem cell-like properties, such as self-renewal, differentiation, and proliferation^10^. These cells are especially dangerous because they can remain dormant and later contribute to metastasis formation^11^. One method of identifying cCSCs is a functional test called the tumorsphere-formation assay that reveals cell proliferation under non-adherent conditions^12^. In this assay, potential cCSCs are characterized by their ability to expand clonally and form tumorspheres in vitro. They can proliferate to form floating 3D spheroids consisting of 30 to 100 cells. The resulting tumorspheres, with the ability to circulate freely in the bloodstream, are characterized by markers such as EpCAM, ALDH1, CD24, CD44, and CD133^13,14^. It has already been shown that the number of tumorspheres correlates with the aggressiveness of the primary tumor such as lymph node involvement and metastasis in several cancer entities^14^. cCSCs in the blood of patients with PDAC have not yet been analyzed^13,14^. Although clinical data is consistent with the hypothesis that CETCs/CTCs contain fractions of cCSCs in PDAC as well, it is necessary to demonstrate their tumorigenic potential in an in vivo model. For this purpose, the chick embryo chorioallantoic membrane (CAM) model provides an excellent method for obtaining patient-derived xenografts (PDXs) from cCSCs.

The CAM is formed during the embryogenesis of the chick embryo and is highly vascularized. It is a versatile, low cost, and reliable model. One key advantage of the CAM model is its multifunctionality since it has been used for various studies on tumor biology, such as angiogenesis, the analysis of tumor metabolism, proliferation and early metastasis to the embryo^15,16^, as well as an individualized drug-testing platform^17^. This in vivo model is widely used since it resembles a model that aligns with the 3R concept of “reduction, refinement, and replacement” of animal experiments^18^. PDXs obtained from cCSCs can be cultured on the CAM for each patient individually, providing the opportunity for both the direct testing of conventional and novel molecular targeted therapeutics, as well as an individualized establishment of precision oncology approaches through molecular analysis.

Therefore, the purpose of this study was twofold: first, to determine the number of CETCs/CTCs and tumorspheres generated from cCSCs and correlate them with clinicopathological parameters in order to evaluate them as clinical biomarkers. Second, to develop a new and personalized preclinical model using PDXs generated from tumorspheres grafted onto the CAM.

## Methods

### Study condition

In this study, the patient population consisted of 25 patients who underwent surgery for PDAC at the University Hospital Regensburg. Patient blood (15 ml) was obtained during a pre-, intra-, and postoperative blood collection via a venous catheter. Intraoperative blood was drawn directly from the portal vein via a venous catheter prior to tumor removal. All blood samples were used to identify and quantify CETCs/CTCs and to generate tumorspheres in vitro^19,20^. Patients gave their informed consent to the research project before surgery. This study was conducted according to the ethical guidelines of the “World Medical Association Declaration of Helsinki- Ethical Principles for Medical Research Involving Human Subjects”. An approval of the ethics committee of the University Hospital Regensburg was also previously obtained (ethics vote 16.05.2022 number 20-1989-101). In addition, the principles of the ARRIVE guidelines were considered in this study to ensure high standards of transparency and reproducibility.

### Origin of CETCs/CTCs and cCSCs

For detection of CETCs/CTCs and cCSCs, the pre-, intra- (portal venous), and postoperative blood samples were collected in EDTA-tubes (ethylenediaminetetraacetic acid) and sent to Laboratory Pachmann in Bayreuth.

#### Detection of CETCs/CTCs

The maintrac® approach was used for counting CETCs/CTCs, as previously reported^19^. After red blood cell lysis, an immunofluorescence assay with a fluorescein-isothiocyanate (FITC)-conjugated EpCAM antibody (clone HEA-125, Miltenyi Biotec, Bergisch Gladbach, Germany) staining was used to identify CETCs/CTCs. Corresponding isotypic control for EpCAM (mouse IgG1k, Miltenyi Biotec, Bergisch Gladbach, Germany) was conducted to determine and subtract background levels of staining and a propidium iodide (PI) staining for the discrimination between living and dead cells was used. Analysis of red and green fluorescence of the cells was performed with a Fluorescence Scanning Microscope, enabling detection and relocation of cells for visual examination of EpCAM positive cells. For data analysis, we used the ScanR Analysis software (Olympus, Hamburg, Germany). Vital CETCs/CTCs were defined as EpCAM-positive cells with intact morphology lacking nuclear PI staining. Only these cells were counted for the analysis (Supplementary Fig. 1a, b).

To improve the characterization of pancreatic circulating tumor cells, Plectin-1 expression was analyzed using an advanced maintrac® approach. For the detection of Plectin-1, an anti-human Plectin-1 phycoerythrin (PE)-conjugated antibody (clone 10F6, Santa Cruz Biotechnology, Dallas, USA) was employed. The isotypic controls for Plectin-1 expression, Mouse IgG PE (Santa Cruz Biotechnology, Dallas, USA), were used at the same final concentration as the primary antibody to ensure specificity and minimize background signals. Additionally, to exclude hematopoietic cells from the analysis, a CD45/pacific blue (clone J33, Beckman Coulter, Brea, USA), staining was performed, allowing for clearer identification of CETCs/CTCs. The results for Plectin-1 were calculated as a percentage of the total number of CETCs/CTCs (Supplementary Fig. 2).

Fluorospheres (Flow-Check 770, Beckman Coulter) enabled the daily verification of optical components and detectors of the microscope to ensure the consistent analysis of samples.

#### Detection of cCSCs

A tumorsphere-formation assay (stemtrac® approach^14^) was used to detect and assess the characteristics of cCSCs. In summary, 1 ml of blood containing circulating cancer cells together with leukocytes were cultured in RPMI-medium supplemented with L-glutamine, HEPES, penicillin/streptomycin, and various growth factors such as EGF, insulin, and hydrocortisone, as previously described^14^. After 14 days of cell culturing, the formation of tumorspheres, indicating the presence of cCSCs, was confirmed under an inverted microscope. Tumorspheres were collected by gentle centrifugation and characterized using a typical combination of markers for pancreatic cancer stem cells. Immunofluorescence staining was performed with FITC-conjugated anti-human EpCAM, PE-conjugated anti-human CD24 (BD Pharmingen™, San Diego, USA), PE-conjugated anti-human CD44 (BD Pharmingen™, San Diego, USA) and PE-conjugated anti-human CD133 (BD Pharmingen™, San Diego, USA). Additionally, tumorspheres were analyzed for the expression of ALDH1 using ALDEFLUOR™ assay (STEMCELL Technologies, Vancouver, Canada) and identified as brightly fluorescent, solid three-dimensional multicellular structures with a diameter greater than 50 µm^14^.

### Patient-derived xenografts on the CAM

**Fig. 1:**
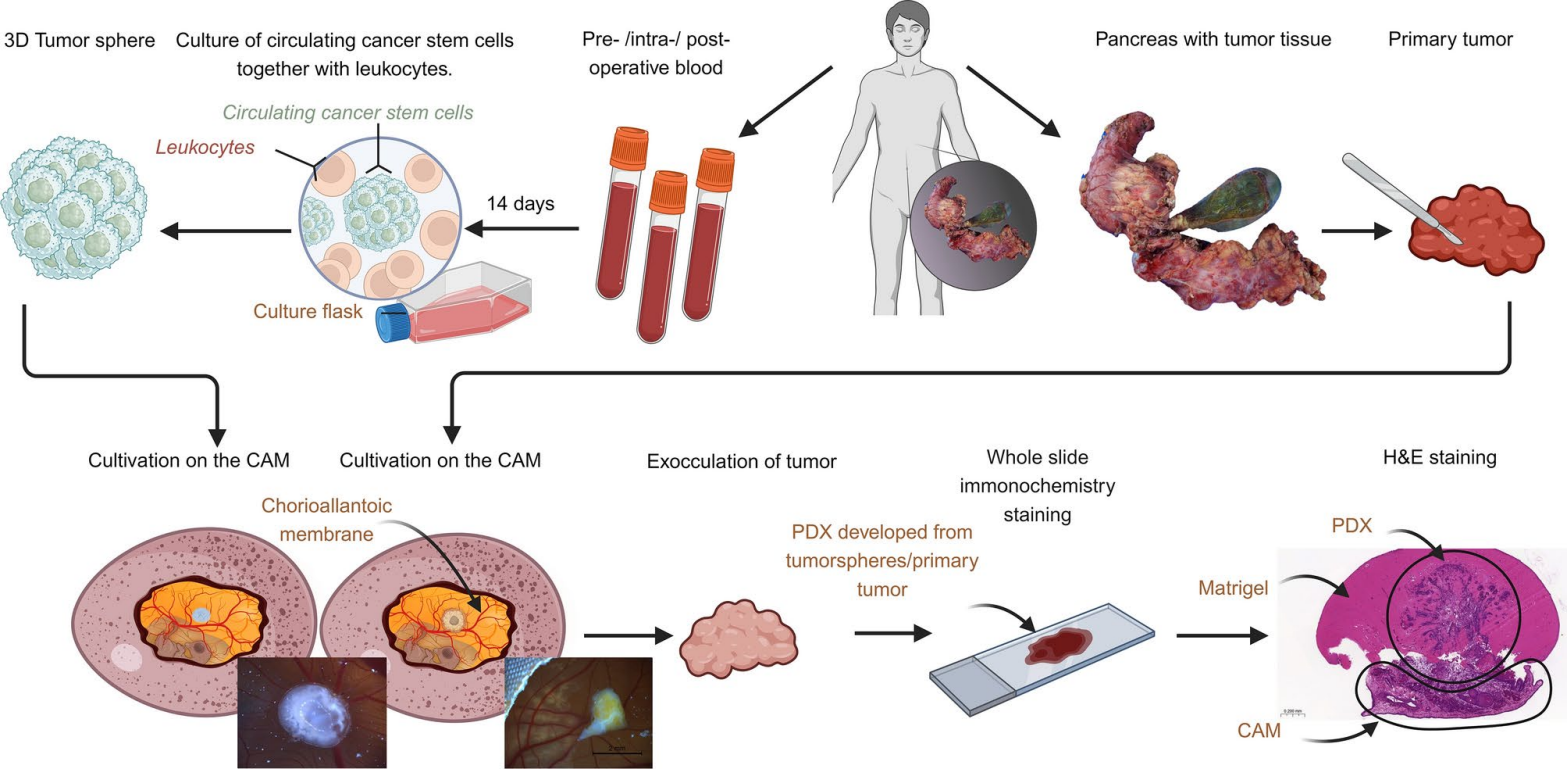
Schematic workflow. Blood samples are used for tumorsphere-formation assay. CAMs are inoculated with patient-derived tumorspheres or samples of the primary tumor. After one week of cultivation on the CAM, the PDX that has grown is exoculated. The PDX is further used for whole slide staining. Figure created with BioRender.com.

The CAM model was performed according to our established and previously published protocol^16,21–23^. Inoculation was carried out with tumorspheres cultured from the patient’s blood or with samples directly derived from the same primary tumor. Tumorspheres were mixed with Matrigel and grafted onto the CAM. Photo documentation was taken. After a period of 7 days, the tumors were explanted and fixed in paraformaldehyde (PFA) 4% and used for whole slide staining (Fig. 1).

### Histopathology

Each PDX, whether it originated from a primary tumor or from tumorspheres, was embedded in paraffin blocks. Subsequently, histological sections were performed and stained with hematoxylin and eosin (H&E). Furthermore, immunohistological stainings were performed with antibodies against p40 (anti-Human p40 monoclonal mouse antibody, clone: BC28 (BioCare)), CD31 (anti-Human CD31 monoclonal mouse antibody, clone: JC70A (DAKO)), Cytokeratin-5/6 (anti-Human Cytokeratin-5/6 monoclonal mouse antibody Clone D5/16 B4 (DAKO)), Cytokeratin-7 (anti-Human Cytokeratin-7 monoclonal mouse antibody, Clone OV-TL 12/30 (DAKO)), and Cytokeratin-19 (anti-Human Cytokeratin-19 LOT 103,069 (PROGEN) monoclonal mouse antibody) (Fig. 1). Histological sections were digitized using a microscopy slide scanner for virtual microscopy (Fritz, Precipoint).

### Statistical analysis and graphs

Statistical analyses were performed using GraphPad Prism Version 8.0.2 (263), released January 30, 2019. A Mann–Whitney U-test was performed to determine statistical differences. For comparison of more than two groups, a Kruskal–Wallis test was performed. In case of significant differences, a Dunn´s Test was conducted to correct for multiple comparisons. *P*-values ≤ 0.05 were considered significant.

## Results

### Clinicopathological characteristics of pancreatic cancer patients

The clinicopathological characteristics of the patients in the present study are summarized in Table 1. The TNM-classification was determined by the corresponding pathology report. In some cases, lymph node involvement or tumor stage was not determined since no curative resection with lymphadenectomy could be performed. In these cases, only a biopsy of peritoneal carcinomatosis or the primary tumor could be used for the analysis. For the same reasons, the M status is not mentioned in every case in the pathology report.

**Table 1:** Clinicopathological characteristics of patients (n = 25) with PDAC.

UICC-Stage	1	2	3	4	Total in %
	20% (5)	44% (11)	16% (4)	20% (5)	100
**Age**					
0–41		9.1% (1)			4
51–60	20% (1)	18% (2)		20% (1)	16
61–70	20% (1)	27% (3)	50% (2)	80% (4)	40
80–100	60% (3)	45% (5)	50% (2)		40
**Sex**					
Female	60% (3)	64% (7)	25% (1)	20% (1)	48
Male	40% (2)	36% (4)	75% (3)	80% (4)	52
**Blood availability**					
Pre-op	100% (5)	100% (11)	100% (4)	100% (5)	100
Intra-op	80% (4)	64% (7)	50% (2)	60% (3)	64
Post-op	100% (5)	91% (10)	100% (4)	60% (3)	88
**T**					
1			25% (1)	20% (1)	8
2	100% (5)	55% (6)	25% (1)	20% (1)	44
3		45% (5)	25% (1)	20% (1)	28
4			25% (1)		4
**N**					
0	100% (5)	18% (2)			28
1		82% (9)		60% (3)	52
2			75% (3)		12
**M**					
0	20% (1)	36% (4)	75% (3)		28
1				100% (5)	20
**R**					
0	60% (3)	82% (9)	100% (4)	20% (1)	68
1	40% (2)	18% (2)		40% (2)	24

### Evidence of circulating cancer stem cells

**Fig. 2:**
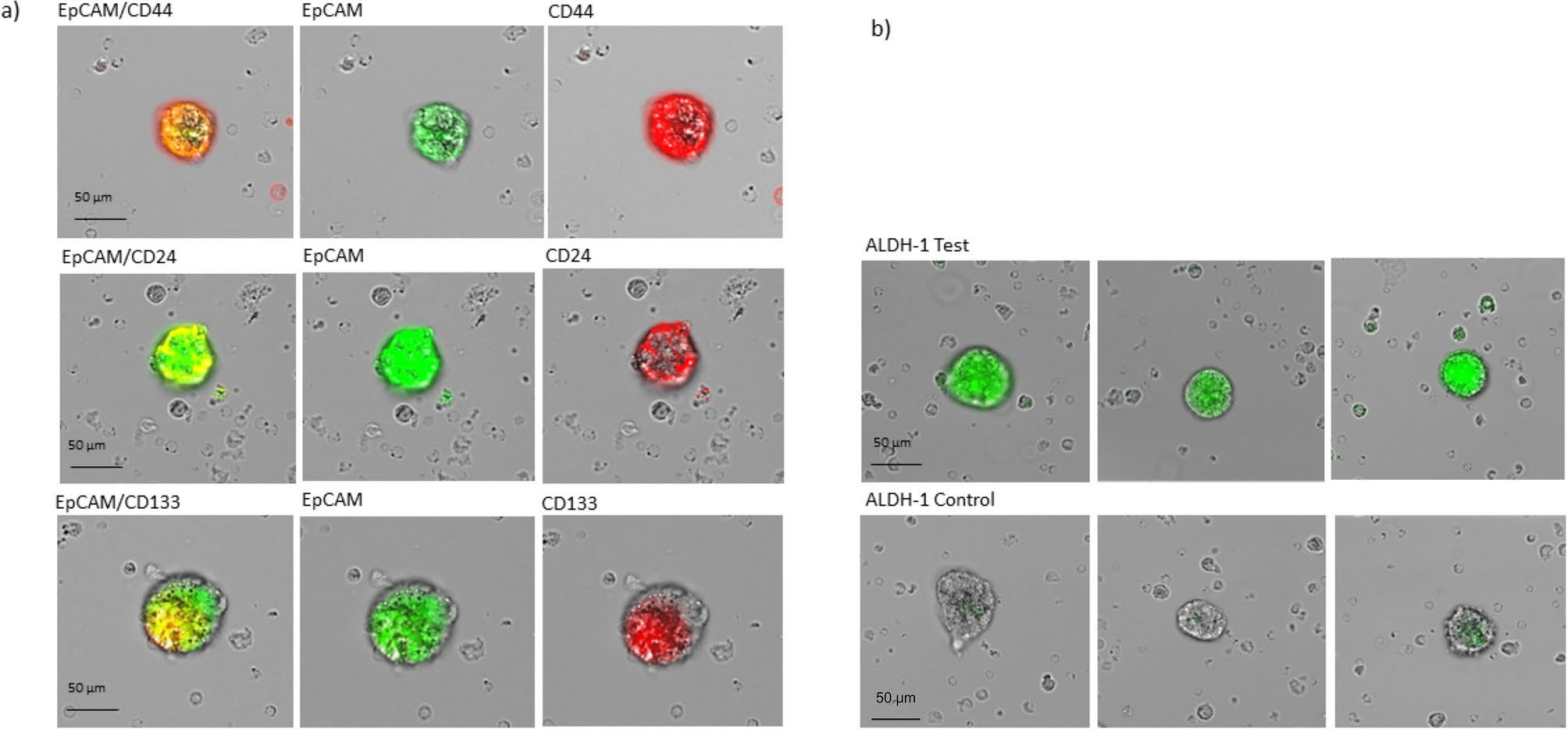
Tumorsphere-formation assay with immunofluorescence staining of tumorspheres. (**a**) Microscopic pictures presenting the morphology of the tumorspheres with spherical colonies of various sizes: Tumorspheres with EpCAM + , CD44 + , CD24 low, and CD133 + staining. (**b**) ALDH-1 activity using ALDEFLUOR™ assay; top: Positive immunofluorescence staining of tumorspheres for ALDH1; bottom: negative ALDH1 control.

Tumor stem cells were detected by growing tumorspheres from blood of pancreatic cancer patients using the stemtrac® approach^14^. Figure 2a shows that after 2 weeks of culture, cCSCs formed spherical colonies (tumorspheres) of various sizes and shapes, derived from the division of a single mother cell. The FITC-conjugated EpCAM antibody (green), PE-conjugated CD44 antibody (red), PE-conjugated CD24 antibody (red), and PE-conjugated CD133 antibody (red) were used to characterize the pancreatic cancer stem cells. The fluorescent immunostaining showed the expression of EpCAM + , CD24 low, CD44 + , and CD133 + by the tumorspheres (Fig. 2a). All tumorspheres exhibited distinct fluorescence for ALDH1 using the ALDEFLUOR™ assay (Fig. 2b), which is characteristic of pancreatic cancer stem cells.

### CETCs/CTCs and cCSCs as clinicopathological markers

**Fig. 3:**
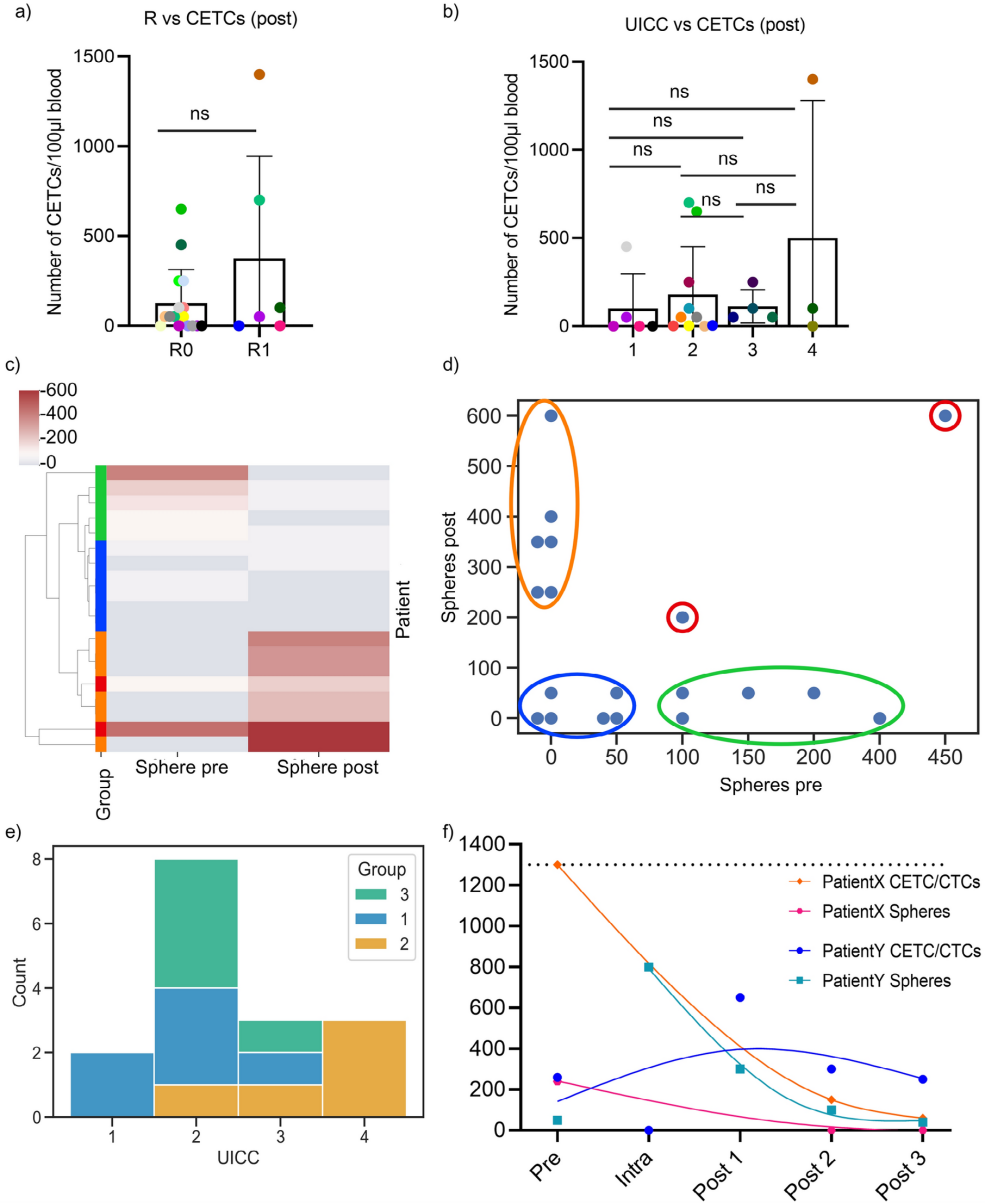
Comparison of CETCs/CTCs (number/100 μl blood) with clinicopathological markers. (**a**) Differences in number of postoperative CETCs/CTCs of patients categorized by their R classification (R0 = complete tumor resection and R1 = remaining cancer cells at the resection margin). (**b**) Differences in number of postoperative CETCs/CTCs of patients grouped by UICC stage (1–4). (**c**) Hierarchical clustering of patients with preoperatively and postoperatively drawn blood according to their tumorsphere values (n = 19). Red values represent a measurement above and blue values represent a measurement below the detected mean value of spheres (86.8/mL blood). (**d**) Scatter plot of the preoperative and postoperative values of the tumorsphere; in case of several patients with the same values, the points were displayed right next to each other in order to avoid overlapping. The three groups were color-coded in blue (group 1), orange (group 2), and green (group 3). The two patients with both pre- and postoperative values above 86.8/mL could not be assigned to any group and were marked in red. (**e**) Distribution of group one, two and three according to their UICC stage in a clustered bar chart (n = 16). (**f**) Time course of CETCs/CTCs and tumorsphere counts for patientX (warm colors) and patientY (cool colors) over the course of therapy, with values for pre-, intra-, and postoperatively (post 1) and during the follow-up (post 2 = follow-up 1, post 3 = follow-up 2).

Clinicopathologic features were compared with the number of CETCs/CTCs and tumorspheres. The highest number of CETCs/CTCs was observed postoperatively in a patient with R1 resection and UICC grade 4. However, the differences in CETCs/CTCs between patients with R0 and R1 resection (*p*-value: 0,5118), as well as between UICC stages (*p*-value: > 0.90), were not statistically significant (Fig. 3a, b).

Additionally, the number of CETCs/CTCs pre- and intraoperatively showed no significant correlation with the UICC-stage (Supplementary Fig. 3).

Plectin-1-positive CETCs/CTCs were observed in all examined patients. The percentage of Plectin-1-positive CETCs/CTCs ranged from 80 to 100% (median, 90%). No association was found between clinicopathological features and the percentage of Plectin-1-positive CETCs/CTCs (Supplementary Fig. 2).

In addition, the number of tumorspheres—a proof that CETCs/CTCs contain fractions of cCSCs that are capable of clonal proliferation—was determined. Here, a subset of 19 patients with both pre- and postoperatively determined number of tumorspheres was arranged via hierarchical clustering (HC) according to their tumorsphere values (Fig. 3c). In this subgroup, the mean value for preoperative tumorspheres was 86.8/mL blood. The HC suggested three different groups: group 1, the number of tumorspheres pre- and postoperatively smaller than the mean (< 86.8/mL) (6 patients); group 2, tumorspheres preoperatively < 86.8/mL and postoperatively > 86.8/mL (6 patients); and group 3, preoperatively > 86.8/mL and postoperatively < 86.8/mL (5 patients). Also, 2 patients could not be assigned to any group (Fig. 3d). For better visualization of the groups, the data is further presented in a scatter plot (Fig. 3d). Finally, group 1, 2 and 3 were correlated with their UICC stage (Fig. 3e). We focused on comparing group 1 and 2. Both groups had low levels before surgical resection, group 1 still had low levels after surgery, while group 2 had high levels of tumorspheres after surgery. The Mann–Whitney-U test resulted in a significant difference between group 1 and 2 (*p*-value: 0.029). Patients in group 1 were distributed in UICC 1, 2, and 3, whereas patients in group 2 had a higher UICC stage (UICC 2, 3 and mainly 4). Group 3 was almost exclusively distributed in UICC 2 apart from one UICC 3 patient.

**Fig. 4:**
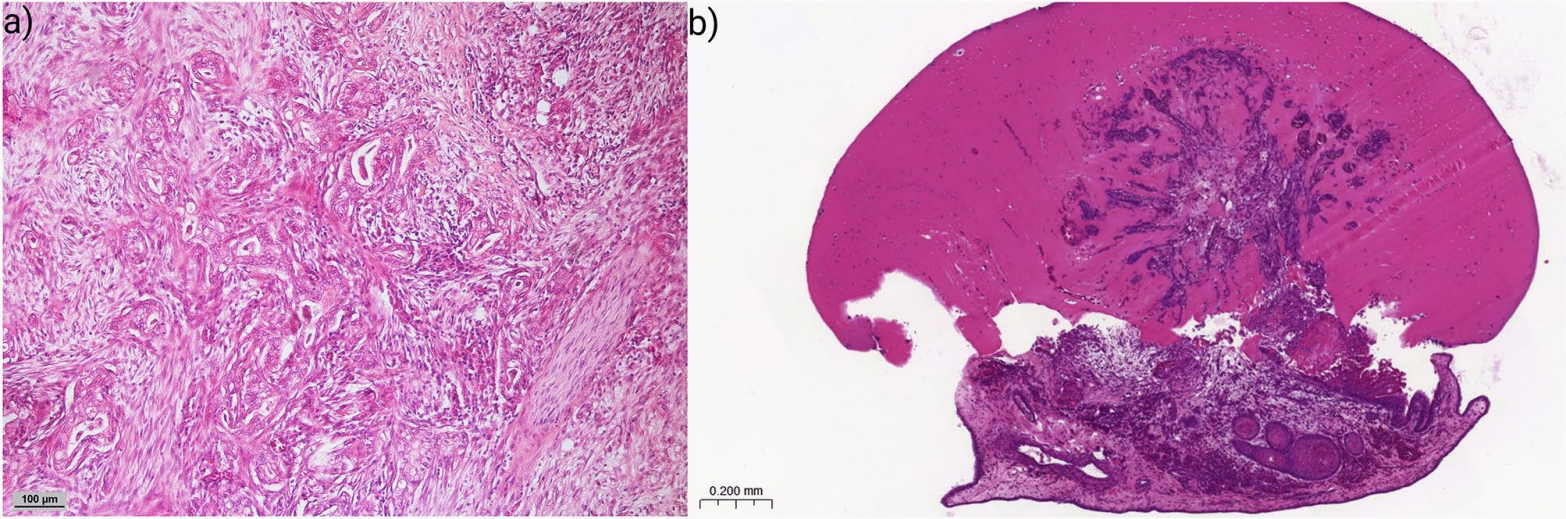
Histological images of patientX. (**a**) Primary pancreatic cancer removed during Whipple procedure. H&E staining. (**b**) Xenograft derived from tumorspheres cultured from patient’s post-surgical blood. H&E staining.

In the following, the case of one of the patients participating in this study, referred to as “patientX”, is presented. PatientX was diagnosed and admitted to the hospital for pancreatic cancer in 2020. The patient had already undergone pancreatic tail resection with splenectomy in 2014 for pancreatic cancer. Clinical analysis of the Computed Tomography (CT) showed a new borderline resectable tumor of the head of the pancreas according to the National Comprehensive Cancer Network guidelines (Supplementary Fig. 4). The portal vein and the superior vena cava showed signs of compression without stenosis or encasement. There was no distant metastasis, direct contact of the tumor with the superior mesenteric and portal vein without stenosis or encasement, as well as no encasement of the common hepatic artery and no extension to the celiac axis. The preoperative screening showed 1300 CETCs/CTCs/mL of blood and 250 tumorspheres/mL of blood (Fig. 3f). After resection of the PDAC through a standard Whipple procedure, the final histopathological diagnosis staged patientX as pT3, L1, V0, R0 (narrow R0), pN1 (2 out of 30 regional lymph nodes affected), M0 and a grading of G2 (UICC 2). After surgery plus six months of adjuvant chemotherapy (mFOLFIRINOX regimen for six cycles, containing folinic acid, fluorouracil, irinotecan and oxaliplatin), 150 CETCs/CTCs and 0 tumorspheres per milliliter blood were detected during a follow-up at 15 months after surgery. 22 months after surgery, the number of CETCs/CTCs decreased to 50/mL, and again no tumorspheres were detected at the second follow-up (Fig. 3f). Also, standard parameters (CEA, CA19-9, contrast-enhanced CT scan) until today showed no signs of recurrence, in particular, no locoregional PDAC growth or distant metastasis (Supplementary Fig. 5). The patient is currently under close surveillance. In Fig. 4a, the primary pancreatic tumor of patientX removed by a Whipple procedure is shown, presenting haphazard ductal proliferation, marked nuclear atypia and desmoplastic stromal reaction.

Another patient, “patientY“, presented with double-duct sign and cholestasis in May 2023, raising suspicion for cancer of the pancreatic head. Preoperative tumor marker levels were 15.4 U/mL for CA 19–9 (reference value: < 27 U/mL) and 6.7 ng/mL for CEA (reference value: < 3.8 ng/mL). Preoperative CETCs/CTCs and tumorspheres were quantified at 250 and 50 per milliliter respectively. Subsequently, the patient underwent the standard Whipple procedure. Intraoperatively, CETCs/CTCs and tumorspheres measurements from the portal vein were 0 CETCs/CTCs/mL but 800 tumorspheres/mL. The final histology reported ductal adenocarcinoma with a TNM classification of pT3, pN0 (0/18), L0, V0, R0 (wide R0), G2. Five days postoperatively, the levels of CETCs/CTCs increased to 650/mL and tumorspheres decreased to 300/mL, respectively. Two months after surgery, adjuvant chemotherapy with mFOLFIRINOX was started. After 6 cycles with dose reduction due to intolerance, a restaging CT revealed stable disease with suspect tissue proliferation alongside the hepatic artery. Measurements of CETCs/CTCs and tumorspheres at this point were 300/mL for both. Following an additional 6 cycles of mFOLFIRINOX with reduced dosage, treatment was terminated, and the final CT indicated no progression of the above-mentioned tissue proliferation or signs of recurrence. Tumor markers returned to normal levels. The last CETCs/CTCs and tumorspheres were measured at 50/mL blood each (Fig. 3f).

### PDXs generated from circulating cancer stem cells using the CAM

In order to prove that tumorspheres are capable of forming real tumors, cCSCs were cultured according to the approach described in the methods section and grafted onto the CAM. Xenografts were successfully generated on the CAM as cell growth across the membrane-tumorsphere border was observed, followed by vascularization and further tumor growth. Thereby the growth rate of tumorspheres on the CAM was 100%. Figure 4b shows a xenograft derived from tumorspheres cultured from postoperative blood withdrawal Supplementary Fig. 6 shows a schematic overview of the workflow of PDX being generated from tumorspheres on the CAM.

**Fig. 5:**
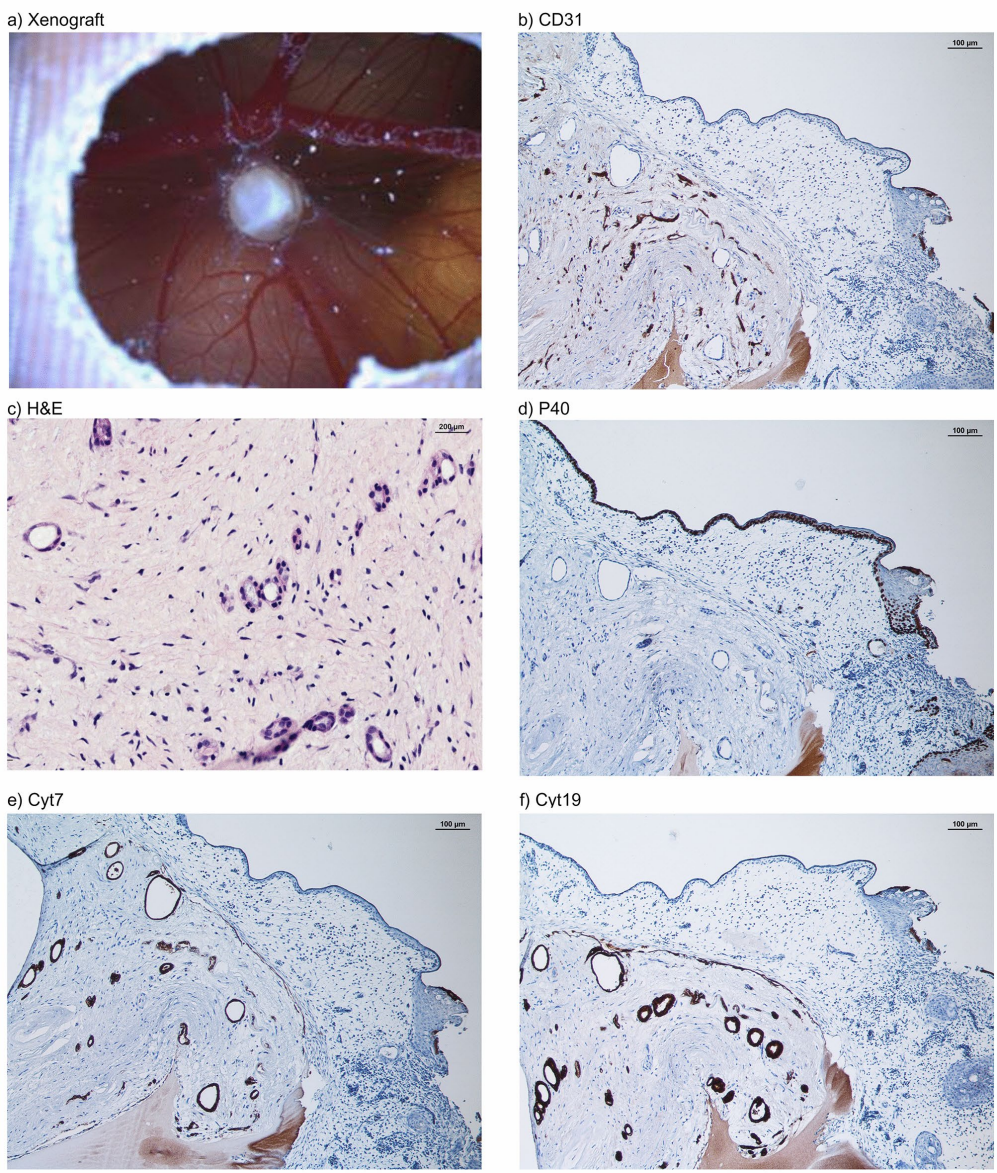
Histological examination of a PDX derived from tumorspheres cultured from patients´ blood postoperatively. (**a**) Macroscopic image of a PDX on the CAM. (**b**) CD31 staining of the CAM PDX developed from tumorspheres with endothelialized vascular proliferation. (**c**) H&E staining of the PDX with ductal epithelial proliferation. (**d**) p40 staining of basal squamous cells. (**e**) Cytokeratin7 staining of adenocarcinoma cells. (**f**) Cytokeratin19 staining of adenocarcinoma cells.

Histological analyses of a PDX shown in Fig. 5a contained neoplastic tissue with characteristic patterns (i.e., comprised of stromal, vascular, and epithelial tissue) comparable to the primary tumor. The CAM tumor was partly distinguishable from the surrounding stroma and the CAM and tumor cells were detected within Matrigel as well as inside the CAM (Fig. 5b, c, e, f). Human CD31, typically used for staining of endothelial cells of the xenograft with ductal epithelial proliferation, resulted in a positive staining of endothelialized vascular structures (Fig. 5b). Further, H&E, Cyt7, and Cyt19 staining revealed solid or dispersed growth of PDAC presenting large, strikingly polymorphous tumor cells surrounding empty lumina of varying size (Fig. 5c, e, f). The outer CAM surface and further basal cell-like structures were positively stained with p40 indicating that the structures originate from chick embryogenesis (Fig. 5d).

## Discussion

PDAC is the fourth leading cause of cancer-related death worldwide. Late or no symptoms and reduced screening possibilities result in delayed diagnosis and a subsequently poor prognosis. Recently, circulating tumor cells have become a promising novel biomarker for hematogenous metastasis of PDAC^24,25,^ but the exact role of circulating tumor cells is still controversial. Many studies indicate that they may be used as a valuable tool for predicting outcomes, identifying personalized treatment and understanding pancreatic tumor biology^26^. Also, new findings have shown that circulating tumor cells can benefit early diagnosis and clinical staging of the PDAC^27–29^. Ankeny et al.^25^, observed a significant correlation between the number of circulating tumor cells and disease progression in preoperative samples. In our perioperative analysis, we did not observe similar findings, potentially due to differences in study design and sample conditions. However, it is important to note that Ankeny et al. only investigated the circulating tumor cells prior to surgery. Recent findings by Tang et al. (2024) have further highlighted the significance of circulating cancer stem cells in PDAC, emphasizing their potential as a diagnostic tool in this context^30^.

In the present study, postoperative CETC/CTC counts were lower in patients with negative resection margins (R0) compared to those with positive resection margins (R1), although this observation was not statistically significant. The elevated CETC/CTC counts observed in patients with R1 resection may be attributable to surgical manipulation or residual microscopic tumor. These findings, however, do not provide statistically reliable evidence for a relationship between resection status and CETC/CTC dynamics and should be interpreted with caution. A similar mechanism might be involved in the increased number of postoperative CETCs/CTCs in higher UICC stages as a PDAC with UICC stage 4 is a more advanced stage in which it is not possible to achieve a R0 resection. This observation requires additional data to confirm its clinical relevance. With more patients enrolled into the ongoing study, the statistical power will increase and following results may reveal more robust insights.

Recently, Plectin-1 has emerged as a promising biomarker for pancreatic ductal adenocarcinoma, with expression spanning from pre-invasive lesions to advanced metastatic stages^31^. Our study also confirmed that circulating tumor cells in the blood of patients with PDAC express Plectin-1 at a very high frequency. In this context, Plectin-1 represents a compelling target for detecting CETCs/CTCs in this group of patients.

A very small fraction of CETCs/CTCs, called cCSCs, which are assumed to be directly responsible for metastasis and recurrence, seem to be of particular interest^14,32^. To prove the metastasis-forming ability of cCSCs and their clonal proliferative potential, a tumorsphere-formation assay was successfully performed and it was able to show positive stainings for EpCAM + , CD24 low, CD44 + , CD133 + , and ALDH1 in spherical colonies formed from a single cancer stem cell (Fig. 2). These markers demonstrate, among others, their stem cell origin, their proliferative capacity, and the capability to initiate tumor progression. cCSCs have been identified in several solid tumors, such as colorectal^33^ and breast cancer^14^. Regarding various gastrointestinal cancers, cCSCs have been linked to an unfavorable prognosis^34,35^. Previously, Pizon et al. observed that the numbers of tumorspheres derived from cCSCs in breast cancer patients correlated with the clinical grading of the tumors^36^. This also appears to be the situation in PDAC. Hierarchical clustering of the patients revealed different groups with group 1, defined by the number of tumorspheres being lower than average (86.8/mL) before and after surgery, and group 2, where the number of tumorspheres was increased postoperatively (Fig. 3c, d). In group 1 patients were associated with a statistically significant lower UICC stage (*p*-value: 0.029) compared to patients in group 2, with higher UICC stages. A potential reason for higher tumorspheres postoperatively in group 2 might be due to a manipulation of tumor tissue remaining in-situ (R1 resection) and/or already existing (micro-) metastasis due to an advanced tumor disease. However, group 3, characterized by higher values preoperatively and lower values postoperatively, mainly clustered in UICC 2, which could be due to a successful resection followed by a rapid decrease of the number of tumorspheres. This indicates that the number of tumorspheres can be used as an additional clinical prognostic biomarker, as it correlates with tumor aggressiveness, as demonstrated by the UICC classification (Fig. 3), providing important information on tumor progression and prognosis. An innovative aspect of this approach is the ability to identify “high-risk constellations” just by collecting blood samples from PDAC patients. These results could present an additional piece of information that could influence clinical decision-making.

In our case report of patientX, the total number of CETCs/CTCs and tumorspheres decreased continuously over the course of treatment, as a sign of successful therapy, which corresponded to the CT scans and the patient’s clinical status. PatientX, classified as UICC 2, had high preoperative levels of CETCs/CTCs and tumorspheres, which decreased after R0 resection to very low levels of CETCs/CTCs and undetectable tumorspheres. A similar outcome was noted in the case of patientY. Despite initial suspicious findings for a recurrence based on the first restaging CT scan, both CETCs/CTCs and tumorspheres exhibited a continuous decrease. This trend persisted and was confirmed by the final CT scan, which described the previously suspicious tissue proliferation as non-suspicious. When considered alongside the overall evaluation of all patients in this study, these individual cases highlight the potential relevance of CETCs/CTCs and tumorspheres, which showed an association with UICC stages, as biomarkers for monitoring treatment response and disease progression. The observed trends in patients X and Y, aligning with clinical and radiological findings, further emphasize their potential for personalized monitoring.

The importance of the finding of tumorsphere formation in peripheral blood as a sign of more aggressive tumors was corroborated by the innovative assay to successfully cultivate tumorspheres on the CAM. The CAM is an ideal xenograft model to study cancer cell invasion and metastasis^15^. The growth efficiency rate of inoculation using PDXs in the CAM model varies widely, ranging from about 40% to 94%^37,38^ depending on the type of PDXs and tumor. Pizon et al. showed a growth efficiency rate of 50% for tumorspheres derived from breast cancer patients. They also associated the growth rate with a higher number of tumorspheres and a high Ki-67 index of the primary tumor^36^. For the first time, we were able to show that tumorspheres derived from cCSCs in patients with pancreatic cancer can induce “tumor-like” growth on the CAM. This was proven by histological evaluation: H&E, Cyt7, Cyt19, and CD31 stainings were used to identify tumor growth characteristics (Fig. 5b, c, e, f). From the pancreatic tumorspheres aberrant pancreatic ducts developed, which is consistent with the histology of human pancreatic ductal adenocarcinoma (Fig. 5b, c, e, f). The PDX tumor glands differed in their appearance from the primary tumor, mainly by lacking the dense desmoplastic stroma. It is widely known that primary tumors often differ from metastases in their differentiation and their stromal component. This might be postulated for PDX tumors as well, since the tumor-surrounding stroma is different from the original pancreatic stroma. Yet the neoplastic and malignant nature of these glands is evident by their increased proliferation and overt nuclear atypia. Furthermore, tumor cells in PDXs were heterogeneously scattered and often detected around blood vessels (Fig. 5e, f) using human CD31 for the staining of structures surrounding the tumor ducts (Fig. 5b). This indicates that tumor cells were not only present next to chicken blood vessels but seem to be able to form human derived blood vessels. A similar phenomenon was described by Ricci-Vitiani ^39^, where orthotopic or subcutaneous injection of glioblastoma stem-like cells in immunocompromised mice produced xenografts with vessel walls formed by human endothelial cells. Bao et al. showed that CSCs produced significantly higher levels of vascular and endothelial growth factor (VEGF) compared to a population of non-CSCs, leading to a long-term angiogenic response^40^. Also, Fujita et al. suggest that CD133 positive CSCs in hepatoblastoma liver cancer differentiate into tumor vascular endothelial cells and might be able to form tumor vessels^41^. In 2022, Benjakul et al.^42^ demonstrated the ability of PDAC cells lines (PANC-1 and MIA-PaCa-2) to engage in a process known as Vasculogenic Mimicry (VM) which causes cancer cells to take on endothelial characteristics by forming tube-like structures. VM has already been shown to be positively corelated to the growth and survival of various solid tumors. Our findings suggest that PDXs stimulated the development of human blood vessels, because VM is not related to histological CD31 staining. Furthermore, previous research on PDAC cell lines has demonstrated that particular growth factors (EGF, VEGF) induce cell differentiation to create tube-like structures^43^. Therefore, we assume that tumorspheres secrete several different growth factors that result in cell differentiation. Future experiments should be conducted to investigate the gene expression pattern of tumorspheres and clarify which growth factors promote PDX-induced cell differentiation. The ability of circulating cancer stem cells to directly contribute to tumor vascularization through endothelial cell differentiation represents a novel mechanism of angiogenesis and may be a potential target for future therapies of pancreatic cancer. Furthermore, the successful creation of PDXs derived from pancreatic cCSCs and primary tumor can enable new treatment possibilities and applications. In the future, the PDXs on the CAM could be used as a continuous follow-up model to monitor and understand the course of therapies, understand the process of metastasis, and enable individualized drug selection in vivo. These therapeutic and diagnostic steps could even be carried out preoperatively through a liquid biopsy.

In addition to the potential applications of PDXs on the CAM for personalized therapy monitoring and metastasis studies, selecting effective drugs remains a critical aspect of improving treatment outcomes. Traditionally, two-dimensional (2D) cell cultures, in which cells grow as a monolayer, have long been the gold standard for in vitro cancer research, especially for studying cellular responses to chemotherapy. These cultures allow uniform access to nutrients and therapeutics across all cells. However, significant drawbacks limit their suitability for studying potential therapies, the most important being their limited resemblance to physiological conditions and the natural cellular microenvironment^44^. Three-dimensional (3D) tissue culture models, including tumorsphere formation followed by inoculation onto the CAM, enable the investigation of cell-to-cell and cell-to-matrix interactions. Cancer cells in 3D cultures exhibit behaviors distinct from those in 2D systems, largely because they preserve key signals from the extracellular matrix (ECM), which is crucial in regulating cellular functions. This is particularly important, as the ECM along with components like its cancer-associated fibroblasts (CAFs) plays a central role in cancer development, progression and treatment resistance^44–48^. In addition, the inhibition of angiogenesis has been an established therapeutic strategy for many solid tumors. However, clinical outcomes for PDAC patients receiving anti-angiogenic therapies remain unsatisfactory. The CAM, with its ability to visualize angiogenesis, provides a valuable tool for research on this topic^49^. With the advantages offered by the CAM, it will be possible to assess the most effective and individualized chemotherapy, particularly by evaluating resistances and efficacies that are not observable in 2D cell cultures^50,51^. With the rise of immunotherapies in PDAC, immune-based studies may even be initiated from day 10 of embryonic development. However, their application on the CAM requires further evaluation, as the immune system of the chick embryo is not fully developed until day 18 and differs in several aspects from the human immune system^52^. Disadvantages, such as the limited service life of the CAM due to ethical considerations and the short embryonic lifespan of 19 to 21 days, must also be taken into account in the application of chemotherapy or immunotherapy. Further studies are essential to refine these approaches, address current limitations, and fully realize the potential of CAM models for advancing personalized chemotherapy and immunotherapy strategies in PDAC treatment.

## Conclusion

This study demonstrated that it is possible to detect circulating tumor cells as well as cells capable of clonal growth into tumorspheres in patients with pancreatic cancer. Although further research is required, the significant findings for tumorspheres as biomarkers, alongside the observed trends for CETCs/CTCs in individual cases, support their combined potential in clinical decision-making to guide individual therapeutic strategies and inform patient prognosis. Furthermore, this is the first time that tumorspheres derived from cCSCs in patients with pancreatic cancer have been observed to induce “tumor-like” growth on the CAM using the described method. The CAM model engrafted with cCSCs may therefore provide an alternative, personalized platform for future studies, combining existing drug therapies with new strategies for diagnosis and therapy.

## Supplementary Information

Supplementary Information.

## Data Availability

The datasets used and/or analyzed during the current study are available from the corresponding author on reasonable request.
