# Supplementary material for: Patient-derived xenografts from circulating cancer stem cells as a preclinical model for personalized pancreatic cancer research
Source: Sci Rep. 2025 Jan 23;15:2896. doi: 10.1038/s41598-025-87054-z (PMC11754431; doi:10.1038/s41598-025-87054-z)

Supplementary file Information:

Title: Patient-Derived Xenografts from Circulating Cancer Stem Cells as a Preclinical Model for Personalized Pancreatic Cancer Research

Journal: Scientific Reports

Authors: Benedikt J. Wagner[a,k,+], Andreas Ettner-Sitter[b,+], Nicolas A. Ihlo[c], Merle Behr[c], Sebastian Kölbl[d], Stefan M. Brunner[a,k], Florian Weber[e], Bettina M. Rau[f], Hans J. Schlitt[a,k], Christoph Brochhausen[g], Rebecca Schönmehl[g], Annalena Artinger[g], Dorothea Schott[h], Monika Pizon[i,h], Katharina Pachmann[i,h], Thiha Aung[b,j], Silke Haerteis[b,#,*], Christina Hackl[a,k,#]

Affiliations: [a]Department of Surgery, University Hospital Regensburg, Franz-Josef-Strauss-Allee 11, 93053 Regensburg, Germany; [b]Institute for Molecular and Cellular Anatomy, University of Regensburg, Universitaetsstrasse 31, 93053 Regensburg, Germany; [c]Faculty of Informatics and Data Science, University of Regensburg, Bajuwarenstrasse 4, 93053 Regensburg, Germany; [d]Technology Campus Hutthurm, Deggendorf Institute of Technology, Hochleiten 1, 94116 Hutthurm, Germany; [e]Institute of Pathology, University of Regensburg, Franz-Josef-Strauss-Allee 11, 93053 Regensburg, Germany; [f] Department of General, Visceral and Thoracic Surgery, Academic Teaching Hospital Neumarkt, Nuernberger Strasse 12, 92318 Neumarkt in der Oberpfalz, Germany; [g]Institute of Pathology, Medical Faculty Mannheim - Heidelberg University, Theodor-Kutzer-Ufer 1-3, 68167 Mannheim, Germany; [h]Simfo GmbH, Kurpromenade 2, 95448 Bayreuth, Germany; [i]Labor Dr. Pachmann Bayreuth, Kurpromenade 2, 95448 Bayreuth, Germany; [j]Faculty of Applied Healthcare Science, Deggendorf Institute of Technology, Dieter-Goerlitz-Platz 1, 94469 Deggendorf, Germany; [k]Bavarian Cancer Research Center (BZKF), University Hospital Regensburg, Franz-Josef-Strauss-Allee 11, 93053 Regensburg, Germany; [+]Authors contributed equally to this work; [#]Authors contributed equally to this work; *Corresponding author

Contact corresponding author: silke.haerteis@ur.de

Supplementary figure 1. Exemplary images of CETCs/CTCs in one patient a) Living CETCs/CTCs with a well-preserved morphology, a bright, green surface EpCAM staining and without red PI nuclear staining. b) Dead CETCs/CTCs with green surface EpCAM staining and red PI nuclear staining.

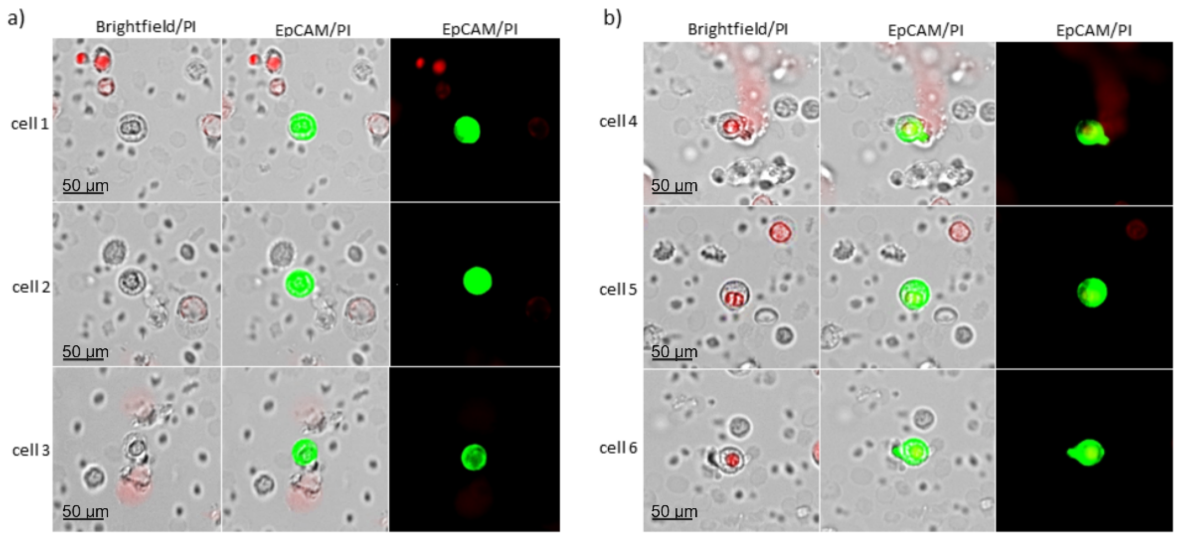

Supplementary figure 2. Identification of CETCs/CTCs in patients with PDAC. Two representative images illustrate PDAC CETCs/CTCs. White blood cells (WBCs) were marked using CD45 staining (blue), while CETCs/CTCs were identified through EpCAM (green) and Plectin-1 (red) staining.

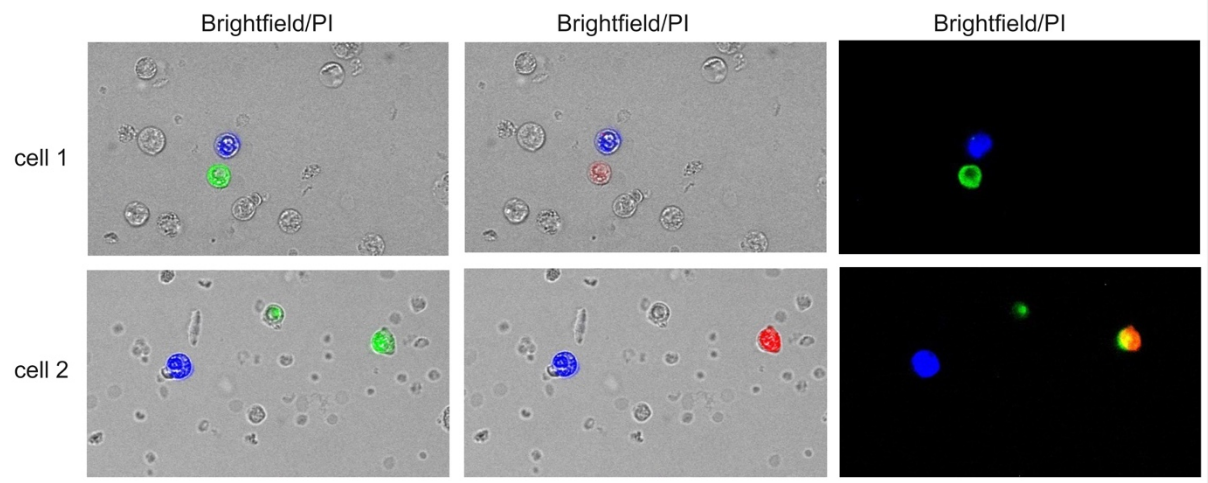

Supplementary figure 3. Comparison of CETCs/CTCs (number/100µl blood) with the UICC grade a) Differences in number of preoperative CETCs/CTCs of patients categorized by UICC stage (p-value > 0,9). b) Difference in number of intraoperative CETCs/CTCs of patients grouped by UICC stage (p-value > 0,9).

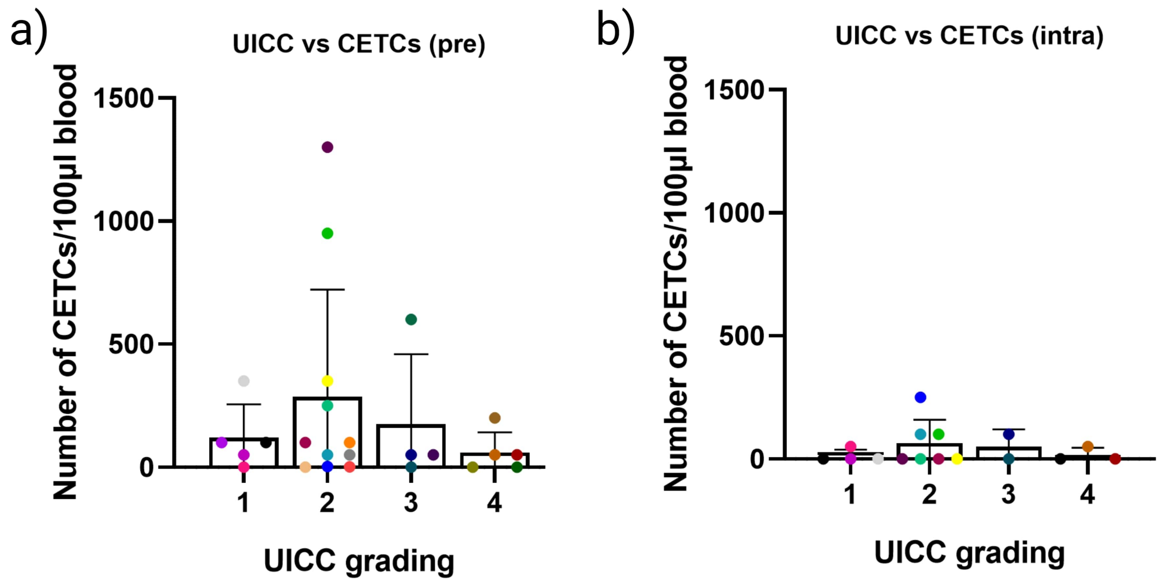

Supplementary figure 4. Computed tomography of pancreatic cancer patientX preoperatively. Arterial phase with contrast agent: a) Sagittal plane b) Axial plane c) Coronal plane; Portal-venous phase: d) Sagittal plane e) Axial plane f) Coronal plane Δ: Superior mesenteric artery, o: Celiac trunk, +: Superior mesenteric vein; p: Portal vein, *PDAC, d: Duodenum.

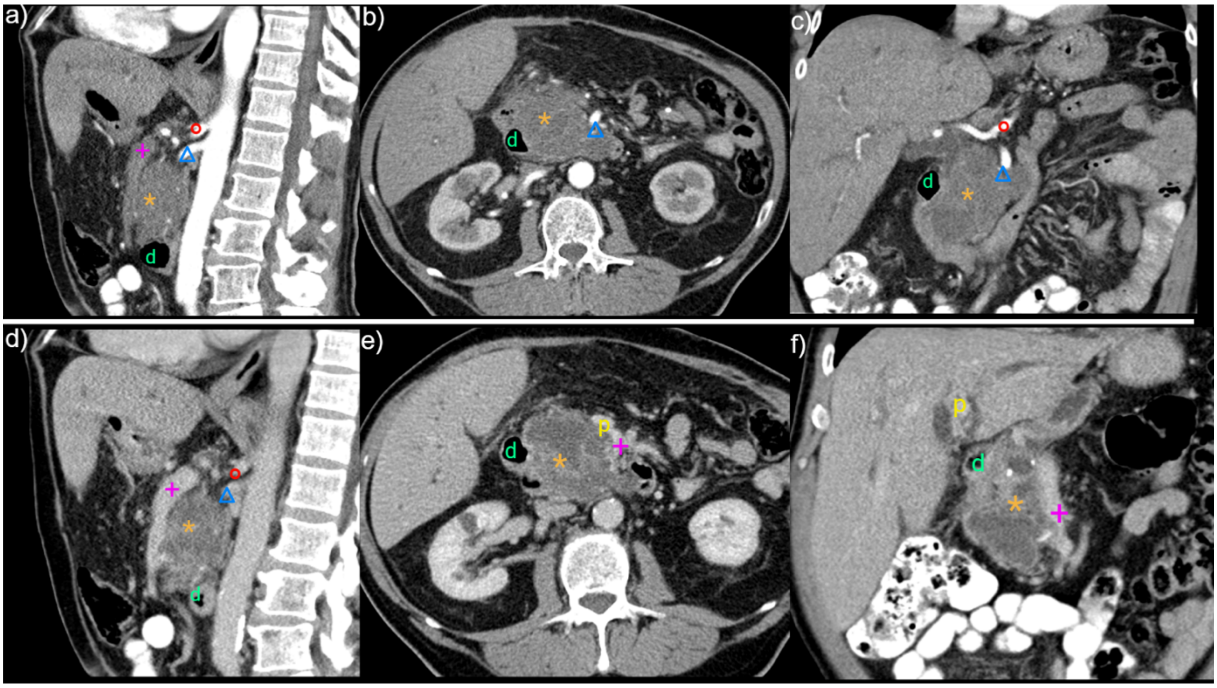

Supplementary figure 5. Computed tomography of pancreatic cancer patientX postoperatively. a) Portal-venous phase, coronal section b) Representative section of patient´s liver in a portal-venous phase without detectable metastasis c) Portal-venous phase, sagittal section d) Representative section of patients´ lung without detectable metastasis Δ: Superior mesenteric artery, o: Celiac trunk, +: Superior mesenteric vein, p: Portal vein.

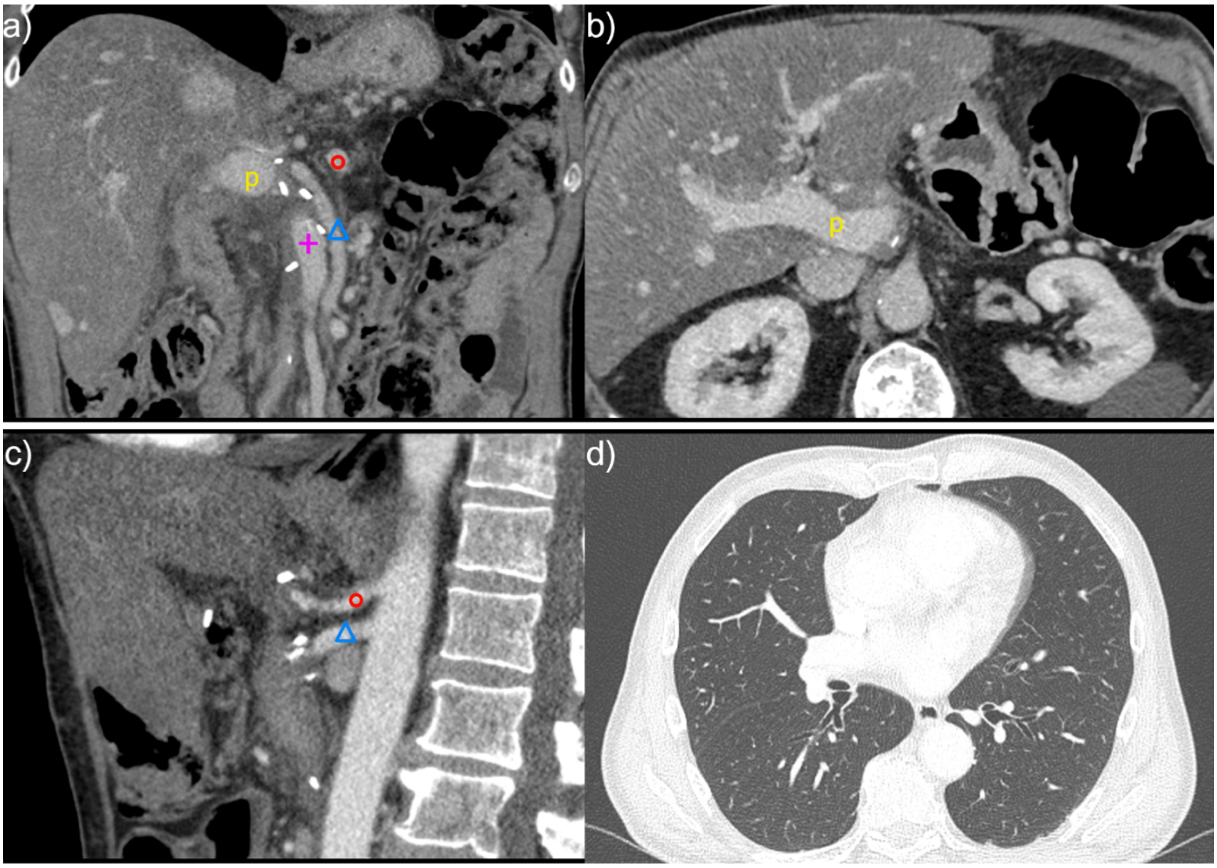

Supplementary figure 6. Schematic overview of patient derived xenografts generated from circulating cancer stem cells on the CAM. The blood of pancreatic cancer patients contains cCSCs that are capable of forming tumorspheres during 21 days in the tumorsphere formation assay. Tumorspheres can be inoculated onto the CAM to detect new tumor development which was detected via H&E and immunohistochemistry. Created with BioRender.com.

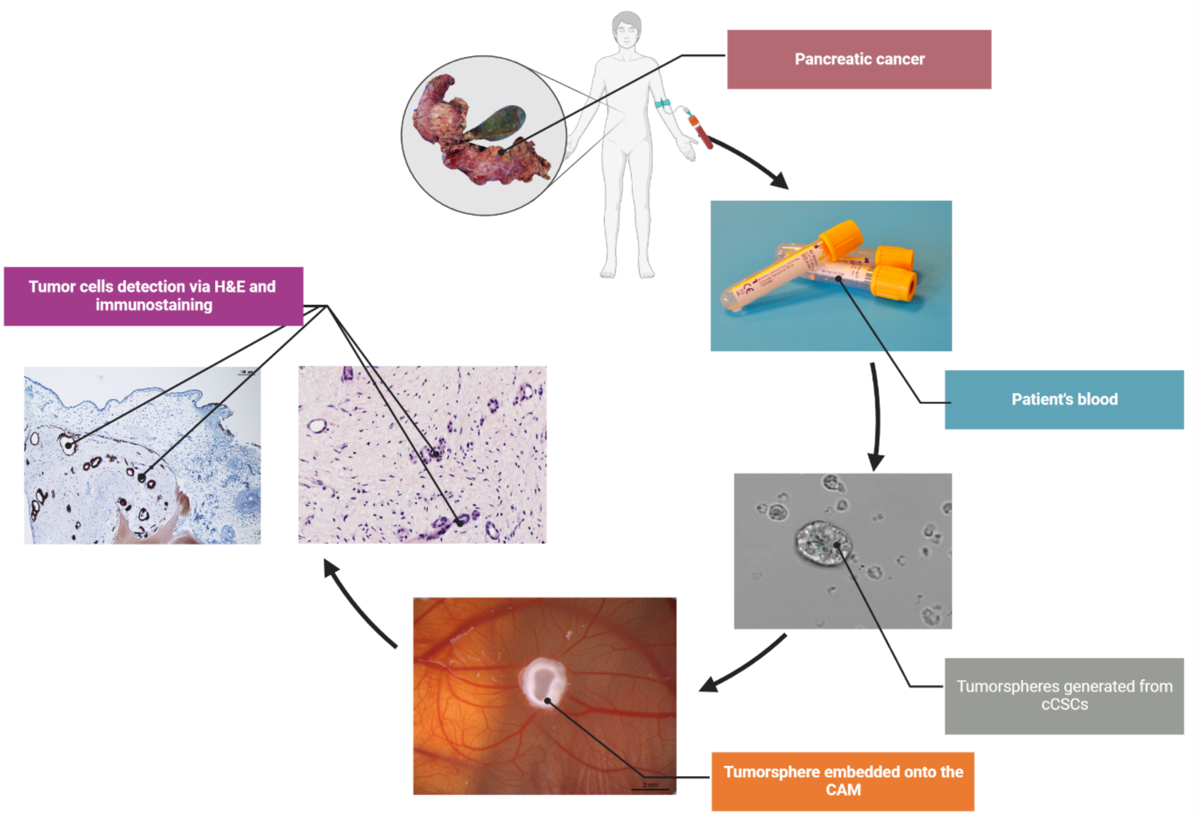

Supplement: Supplementary file 1 — Supplementary Information. [file 41598_2025_87054_MOESM1_ESM.pdf]
